# Individual ingredients of NP-101 (Thymoquinone formula) inhibit SARS-CoV-2 pseudovirus infection

**DOI:** 10.3389/fphar.2024.1291212

**Published:** 2024-02-06

**Authors:** Abdelrahim Maen, Betul Gok Yavuz, Yehia I. Mohamed, Abdullah Esmail, Jianming Lu, Amr Mohamed, Asfar S. Azmi, Mohamed Kaseb, Osama Kasseb, Dan Li, Michelle Gocio, Mehmet Kocak, Abdelhafez Selim, Qing Ma, Ahmed O. Kaseb

**Affiliations:** ^1^ Section of GI Oncology, Houston Methodist Neal Cancer Center, Houston, TX, United States; ^2^ Weill Cornell Medical College, New York, NY, United States; ^3^ Cockrell Center for Advanced Therapeutic Phase I Program, Houston Methodist Research Institute, Houston, TX, United States; ^4^ Department of Gastrointestinal Medical Oncology, The University of Texas MD Anderson Cancer Center, Houston, TX, United States; ^5^ Codex BioSolutions Inc., Rockville, MD, United States; ^6^ Seidman Cancer Center, Case Western University, Multidisciplinary NET Treatment, Cleveland, OH, United States; ^7^ School of Medicine, Wayne State University, Detroit, MI, United States; ^8^ Novatek Pharmaceuticals, Inc., Houston, TX, United States; ^9^ Department of Hematopoietic Biology and Malignancy, University of Texas MD Anderson Cancer Center, Houston, TX, United States; ^10^ Department of Biostatistics and Medical Informatics, International School of Medicine, Istanbul Medipol University, Istanbul, Türkiye; ^11^ Philadelphia College of Osteopathic Medicine (PCOM), Philadelphia, PA, United States

**Keywords:** COVID-19, Thymoquinone TQ, Coronavirus, COVID-19 and anti-viral agents, TQ formula, fatty acids

## Abstract

Thymoquinone TQ, an active ingredient of Nigella Sativa, has been shown to inhibit COVID-19 symptoms in clinical trials. Thymoquinone Formulation (TQF or NP-101) is developed as a novel enteric-coated medication derivative from Nigella Sativa. TQF consists of TQ with a favorable concentration and fatty acids, including palmitic, oleic, and linoleic acids. In this study, we aimed to investigate the roles of individual ingredients of TQF on infection of SARS-CoV-2 variants *in-vitro*, by utilizing Murine Leukemia Virus (MLV) based pseudovirus particles. We demonstrated that NP-101, TQ, and other individual ingredients, including oleic, linoleic, and palmitic acids inhibited SARS-CoV-2 infection in the MLV-based pseudovirus model. A large, randomized phase 2 study of NP-101 is planned in outpatient COVID-19 patients.

## 1 Introduction

The novel COVID-19 virus has had a worldwide impact with more than 500 million cases and around 6 million deaths (WHO, 2022). The Food and Drug Administration (FDA) authorized the use of several medications for both inpatient and outpatient settings (Emergency Use Authorization, 2022). Many compounds have been tested for their activity against COVID-19, including herbal medicines with defined safety and tolerability profiles (Abdelrahim et al., 2022).

**FIGURE 1:**
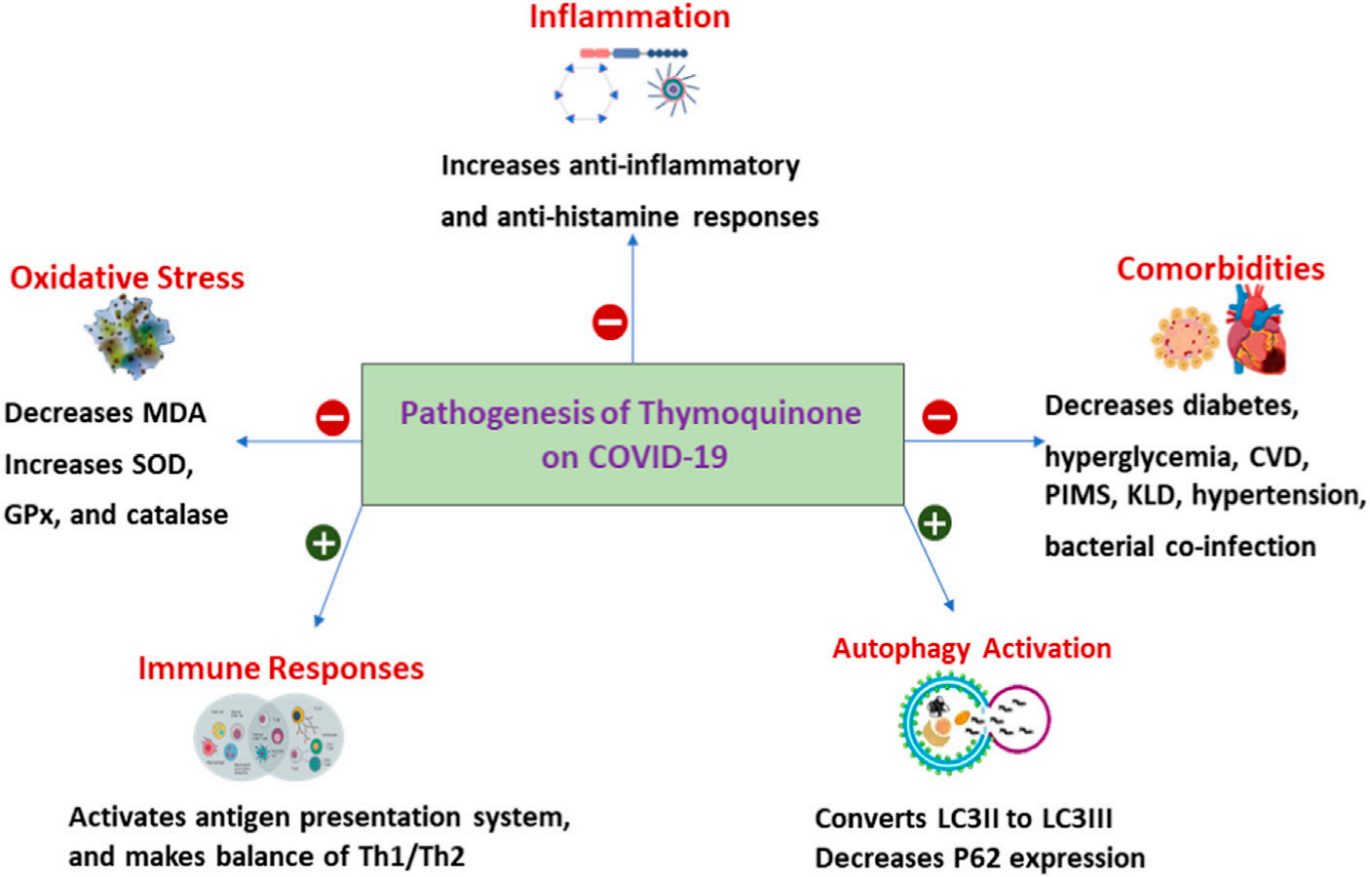
Pathogenesis *Thymoquinone* on COVID-19. SOD, Superoxide dismutase; GPx, Glutathione peroxidase; MDA, Malonaldehyde Th1, type I helper T lymphocytes; Th2, type II helper T cells; CVD, Cardiovascular disease; PIMS, Paediatric Inflammatory Multisystem Syndrome; KLD, Kawasaki-like diseases; LC3, Microtubule-associated protein 1A/1B-light chain 3; P62, protein 62.

*Nigella Sativa*, also known as black seed oil, has been tested in different disease settings as a safe, complementary, and alternative medicine that demonstrated promising anti-inflammatory and anti-tumor effects (Mostofa et al., 2017; Pop et al., 2020). Importantly, *Nigella Sativa* has been identified as a capable medicine against COVID-19. Its use was associated with a higher chance of resolution of symptoms and faster recovery in adult patients with mild COVID-19 (Koshak et al., 2021a). The active ingredient [Thymoquinone (TQ)] of *Nigella Sativa* was proposed to inhibit COVID-19 virus infection by blocking the angiotensin-converting enzyme 2 (ACE2) receptor with immunomodulatory activities (Figure 1) (Rahman, 2020).

TQ formulation (TQF or NP-101) is a novel, patent-pending, enteric-coated formulation that consists of TQ with a tight concentration and fatty acids: palmitic, oleic, and linoleic acids. In a recent randomized, double-blind, placebo-controlled, multicenter phase II trial, oral TQF has been shown to be safe and was associated with a significantly faster decline in the total symptom burden (TSB) in patients with COVID-19 than placebo (Bencheqroun et al., 2022). Notably, TQF has also significantly increased CD4^+^ and CD8^+^ cytotoxic and helper T lymphocytes (Bencheqroun et al., 2022).

In this study, we aim to investigate the impact of the main ingredients of TQF on SARS-CoV-2 infection. For that, we used Murine Leukemia Virus (MLV) based pseudovirus particles that were generated in Human embryonic kidney (HEK) 293T cells with a SARS-CoV-2 spike protein construct. We tested the individual components of TQF on SARS-CoV-2 pseudovirus infection by using ACE2 expressed-HEK293T. We showed that not only TQ but also other individual ingredients of NP-101, such as palmitic, oleic, and linoleic acids had inhibitory effects on SARS-CoV-2 variants with various efficacy, possibly by inhibiting viral entry via the ACE-2 receptor.

## 2 Materials and methods

Cell lines, constructs, and pseudoviruses. HEK293T was obtained from ATCC (American Type Culture Collection, Cat# CRL-3216) (Manassas, VA). HEK293T, stably overexpressing the hACE2 receptor, was obtained from Codex BioSolutions (Gaithersburg, Maryland). Both cell lines were maintained in Dulbecco’s MEM (Cat# 25-500, Genesee Scientific) containing 10% fetal bovine serum (Cat# 35-010-CV, Corning Life Sciences). The plasmid map of the SARS-CoV-2-Spike expression vector is shown in Supplementary Figure S1.

### 2.1 Test compounds

TQF (NP-101) was received from Novatek Pharmaceuticals. TQ (Cat# 03416) was purchased from MilliporeSigma. Oleic acid (Cat# O1008), Linoleic acid (Cat# L1376), and Palmitic acid (Cat# P0500) were purchased from MilliporeSigma.

The preclinical work was performed by Codex BioSolutions Inc, Gaithersburg, Maryland.

### 2.2 Generating of SARS-CoV-2 pseudo particles

MLV particles pseudotyped with a SARS-CoV-2 spike protein construct was generated in HEK293T cells, following a protocol described previously for SARS-CoV with some modification (Mill et al., 2016; Chen et al., 2020; Xiao et al., 2021). All the plasmid DNAs were purified with ZymoPURE II Plasmid Midiprep Kit (Cat# D4201, Zymo Research). In brief, 8 million HEK293T cells were plated into a 10-cm tissue culture dish (Cat# sc-251460, Santa Cruz) in 16 mL DMEM (Cat# 25-500, Genesee Scientific) +10% FBS (Cat# 35-010-CV, Corning Life Sciences) without any antibiotics. On the second day, the cells were transfected with 8 μg pTG-Luc, 6 μg pCMV-MLVgag-pol, and 6 μg pcDNA3.1-SARS-CoV-2-Spike- ∆C19 of different variants (Supplementary Figures S2–S5) using Lipofectamine 3,000 reagent (Cat# L3000015, Thermo Fisher). The cells were cultured for an additional 48 h. The supernatant was collected into a 50-mL Falcon tube and spun at 290 × g for 7 min. The supernatant (pseudotyped virus solution) was then passed through a 0.45 μm filter (Cat# sc 358814, Santa Cruz) using an appropriate syringe. The pseudotyped virus solution was aliquoted into cryovials and stored at −80°C. Each 10-cm cell culture dish produces about 16 mL SARS-CoV-2-PP. The SARS-CoV-2-PP was tested for quality control with the HEK293-ACE2 cell line. The quality controls were performed by two different methods: 1) RNAs in the PP were extracted with Takara’s viral RNA/DNA purification kit (Cat# 740983.50). qRT-PCR was then performed with ThermoFisher’s Power SYBR Green RNA-to-CT 1-Step Kit (Cat# 4389986) on QuantStudio 3 Real-Time PCR Systems. *In Vitro* transcribe luciferase RNA was used a control. The titer was calculated for each PP; 2) Each lot of PP was tested by infecting HEK293 cells. After 42-h infection, luciferase activities were measured with Codex’s Luciferase assay reagent (CB-80552-010).

### 2.3 Testing of SARS-CoV-2 pseudovirus infection by the test compounds

In brief, 7.5 × 103 HEK293 cells, stably transfected with a full-length human ACE2 expression construct in a 15 µL culture medium, were plated into a 384-well white-clear plate coated with poly-D-Lysine to enhance the cell attachment. On Day 2, 12.5 µL of SARS-CoV-2 MLV pseudoviruses for each variant were mixed with 5 µL of each compound at different concentrations and incubated at 37°C for 30 min. After the medium in each well containing the cells was removed, 17.5 µL of each compound-virus mixture was added. The plate was centrifuged at 54 × g for 15 min at 4°C, and an additional 7·5 µL of culture medium was then added. The final total volume in each well was 25 µL. The cells were then incubated at 37°C for 42 h. Luciferase activities were measured with a Firefly Luciferase Assay Kit (CB-80552-010, Codex BioSolutions Inc). At the same time, the cell toxicities of the fatty acids on HEK293-ACE2 cells were tested using the Codex’s EnerCount cell growth assay kit, which measures the ATP levels inside the cells (Cat# CB-80551-010, Codex BioSolutions). The data were normalized as the percentage of the highest reading (low concentration of each compound or no compound) of each compound (relative luciferase activity, RLU). These data were used to draw the dose-response curves against the compound concentrations. IC50 values were calculated based on curve fitting in GraphPad Prism.

## 3 Results

**FIGURE 2:**
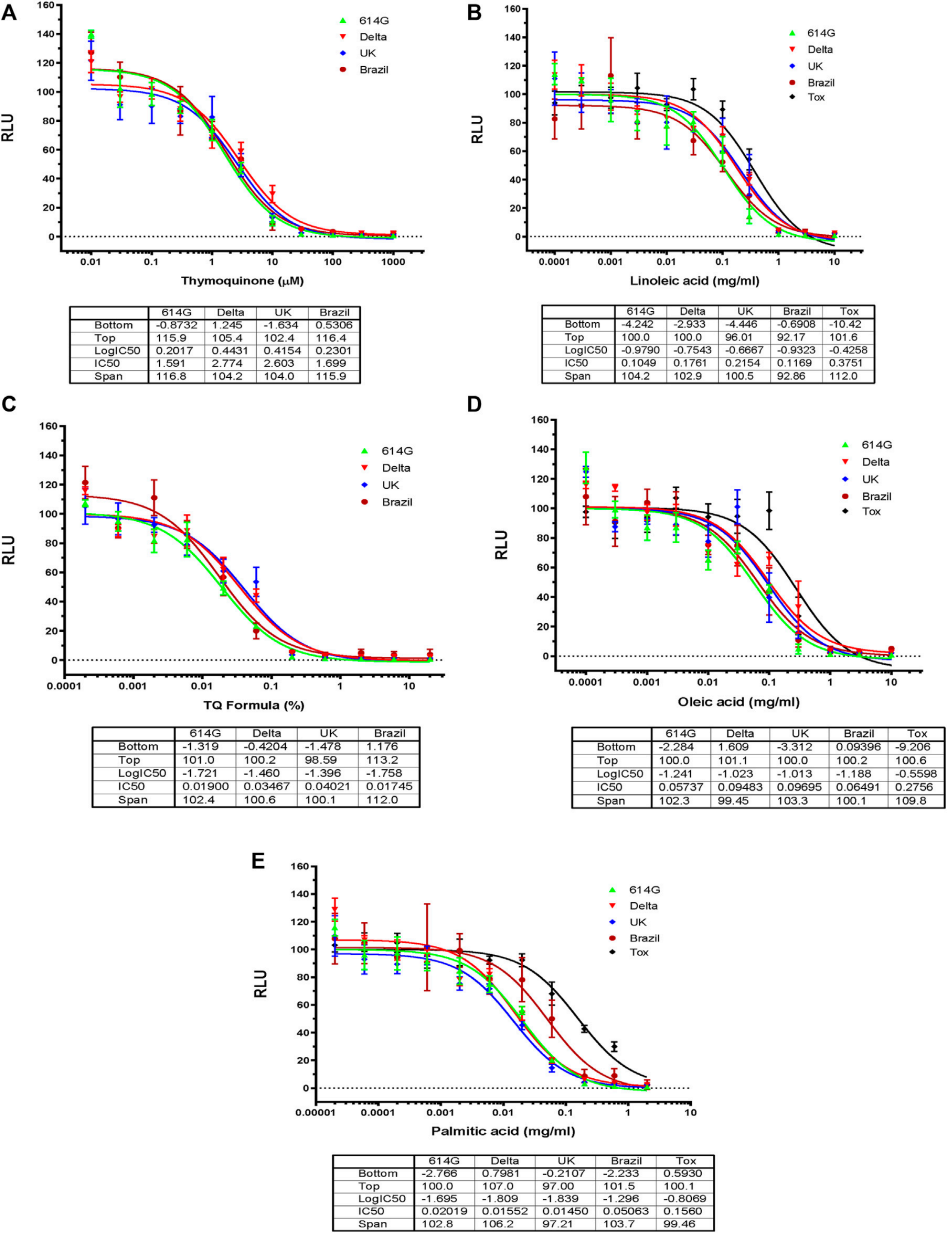
Effects of Blackseed oil, Thymoquinone TQ and Fatty Acids on Blocking SARS-CoV-2-PP Infection of ACE2-expressing Cells. Serial dilutions of Blackseed oil**,** Thymoquinone TQ**,** Oleic acid**,** Linoleic acid**,** and Palmitic acid were tested for inhibition against an MLV-based pseudotyped virus using four different SARS-CoV-2 variant spike protein constructs (614D, Delta, United Kingdom, Brazil), in the infection of HEK293-ACE2 cells. The cell toxicities of the fatty acids on HEK293-ACE2 cells were shown as ‘Tox’. *X*-axis: compound concentration. The *Y*-axis shows relative luminescence unit (RLU), reflecting the luciferase activity and the viral infectivity. The experiments were repeated three times with independent samples giving similar results. For all panels, the data points shown are mean and s.d. for n = 3 technical replicates. IC50 values derived from curve fitting are listed in the Table below each Graph.

TQF is the first-ever enteric-coated capsule derived from *Nigella Sativa* oil. It is characterized and manufactured under Good Manufacturing Practices (GMP) and its patent application is currently under process (Bencheqroun et al., 2022). It consists of TQ and fatty acids, including palmitic oil, linoleic, and oleic acids. We have previously shown that both black seed oil and TQ had inhibitory effects on all four SARS-CoV-2 variants (614D, Delta, United Kingdom, Brazil) with an IC50 value range between 0.01% and 0.04% and 1.5 mM–3EmM, respectively (Figures 2A, C) (Bencheqroun et al., 2022). In this study, by using SARS-CoV-2 eGFP-luciferase spike protein pseudo-viruses, we evaluated the effect of other ingredients of the TQ formula including oleic, linoleic, and palmitic acids on viral entry into HEK293T cells stably overexpressing the hACE2 receptor (Figures 2A, C). These fatty acids also showed an inhibitory effect on infection of all four SARS-CoV-2 variants individually with an IC50 value range between 0.05 and 0.1 mg/mL for oleic acid (Figure 2D), 0.1 to 0.2 mg/mL for linoleic acid (Figure 2B), and 0.02 to 0.05 mg/mL for palmitic acid (Figure 2E). We also tested the toxicity of fatty acids on HEK293-ACE2 cells by utilizing Codex’s EnerCount cell growth assay. IC50 values for cell cytotoxicity were 0.3 mg/mL for oleic acid, 0.4 mg/mL for linoleic acid, and 0.2 mg/mL for palmitic acid (Figure 2E).

**FIGURE 3:**
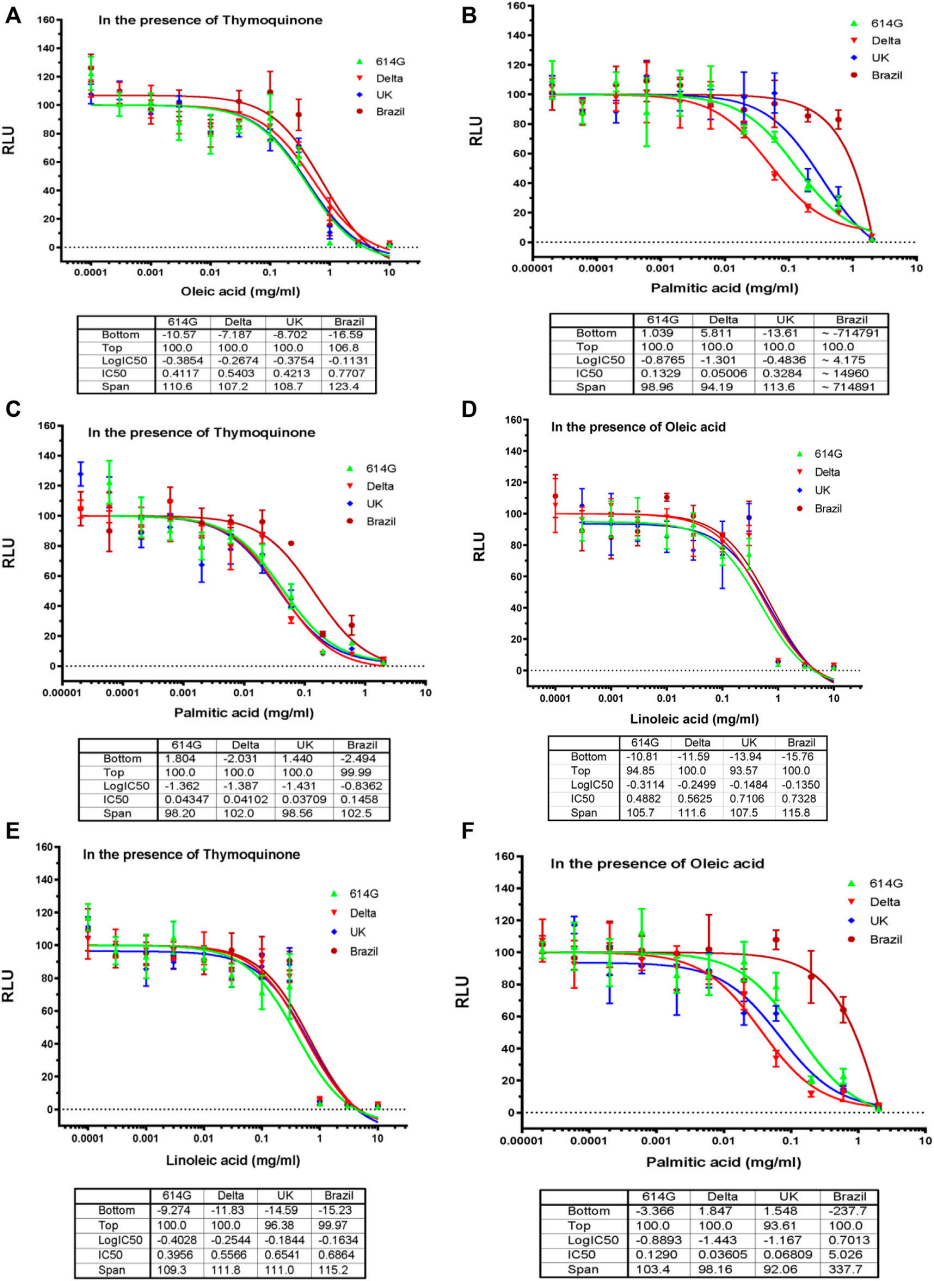
Effects of Oleic acid, Linoleic Acid, and Palmitic Acid in Combination with TQ or with Another Fatty Acid on Blocking SARS-CoV-2-PP Infection of ACE2-expressing Cells. Serial dilutions of oleic acid**,** linoleic acid and palmitic acid in the presence of 0.05 μM TQ; serial dilutions of linoleic acid and palmitic acid in the presence of 0.01 mg/mL oleic acid; serial dilutions of palmitic acid in the presence of 0.01 mg/mL linoleic acid were tested for inhibition against an MLV-based pseudotyped virus using four different SARS-CoV-2 variant spike protein constructs (614D, Delta, United Kingdom, Brazil), in the infection of HEK293-ACE2 cells. *X*-axis: compound concentration. *Y*-axis: relative luminescence unit (RLU), reflecting the luciferase activity and the viral infectivity. The experiments were repeated three times with independent samples giving similar results. For all panels, data points shown are mean and s.d. for n = 3 technical replicates. IC50 values derived from curve fitting are listed in the Table below each Graph.

Based on the results in Figure 2, we decided to perform dose-response assays for oleic acid, linoleic acid, and palmitic acid in the presence of 0.05 mM TQ; dose-response assays for linoleic acid and palmitic acid in the presence of 0.01 mg/mL of oleic acid; dose-response assays for palmitic acid in the presence of 0.01 mg/mL linoleic acid. Fatty acids showed an inhibitory effect on SARS-CoV-2 variants in the presence of 0.05 mM TQ with an IC50 value range between 0.4 and 0.8 mg/mL for oleic acid (Figure 3A), 0.4 to 0.7 mg/mL for linoleic acid (Figure 3 E), and 0.04 mg/mL to 0.1 mg/mL for palmitic acid (Figure 3C). Linoleic acid and palmitic acid appeared to inhibit SARS-CoV-2 variants in the presence of 0.01 mg/mL oleic acid with IC50 values ranging between 0.5 and 0.7 mg/mL and 0.04 to 5 mg/mL (Figures 3B,D,F) respectively. Linoleic acid also inhibited three SARS-CoV-2 variants (Delta, 614G, United Kingdom) in the presence of 0.01 mg/mL palmitic acid with an IC50 value range between 0.05 and 0.3 mg/mL.

**FIGURE 4:**
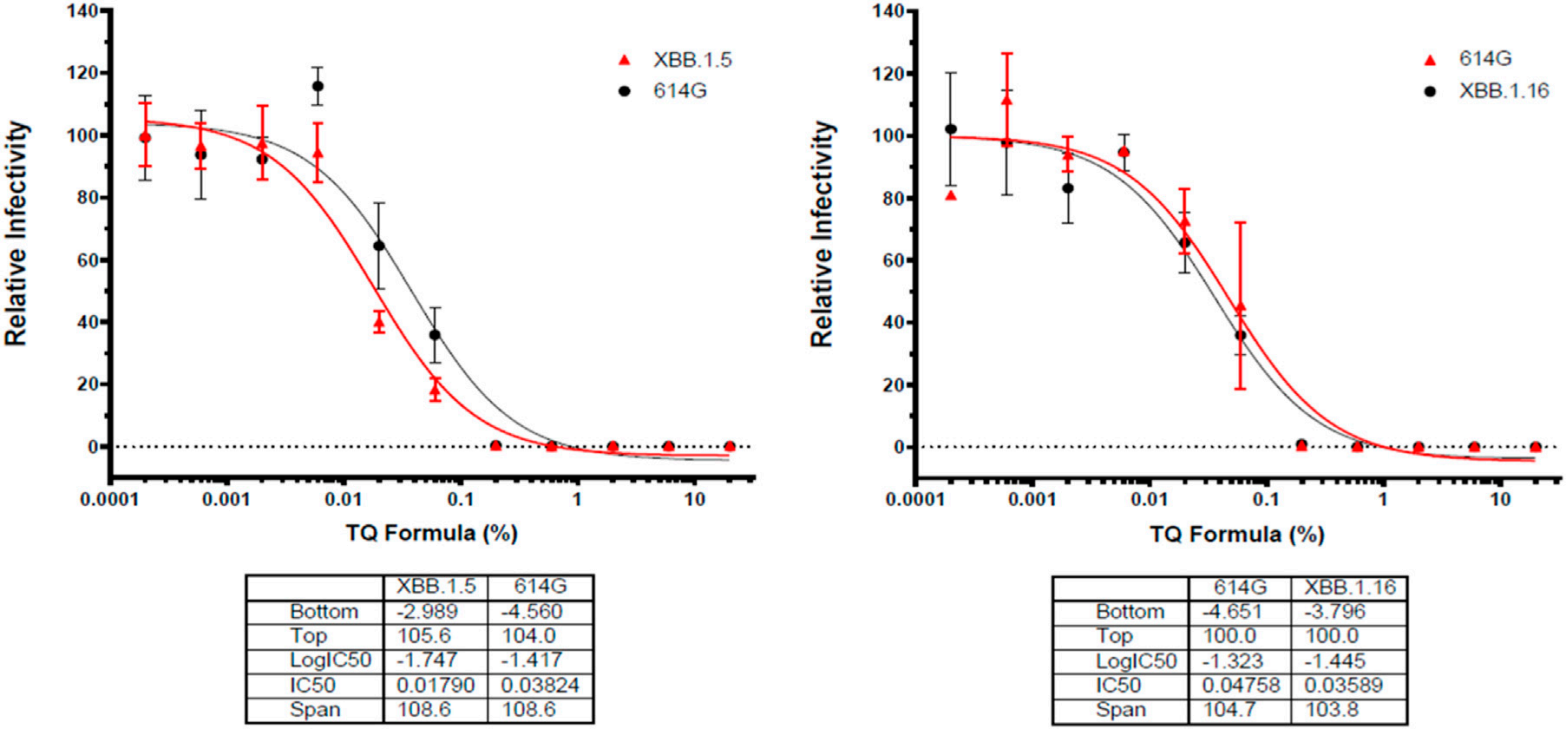
Effects of TQ Formula in percentage increments in relativity to the infectivity of Sars-CoV-19 subvariants XBB.1.5 (Omicron) and 614G. Serial dilutions of oleic acid, linoleic acid and palmitic acid in the presence of 0.05 μM TQ; serial dilutions of linoleic acid and palmitic acid in the presence of 0.01 mg/mL oleic acid; serial dilutions of palmitic acid in the presence of 0.01 mg/mL linoleic acid were tested for inhibition against an MLV-based pseudotyped virus using four different SARS-CoV-2 variant spike protein constructs (614G and Omicron) in the infection of HEK293-ACE2 cells. *X*-axis: compound concentration. *Y*-axis: relative infectivity of viral component. The experiments were repeated three times with independent samples giving similar results. For all panels, data points shown are mean and s.d. for n = 3 technical replicates. IC50 values derived from curve fitting are listed in the Table below each Graph.

Additionally, the sudden rise in prominence of subvariants such as 614G and XBB.1.5 (Omicron) made TQ formulation dose-response assays for relative effectiveness appropriate (Figure 4). The results demonstrated after a 0.01 dose of TQ formulation there was a substantial decline in the infectivity of both observed subvariants (Figure 4). Moreover, infectivity shows such a decline that both pseudo-viruses 614G and Omicron potency had touched zero by the time TQ levels reached a one percent formula (Figure 4).

## 4 Discussion

Oral drug therapy is urgently needed for the treatment of mild to moderate SARS-CoV-2 infections in outpatient settings for 2 main reasons: 1) to enhance disease recovery and shorten the time to resolution of symptoms, and 2) to prevent progression to severe disease and hospitalization or death**.** Herbal medications have been getting attention in the COVID-19 era, given their favorable safety and tolerability profiles. *Nigella Sativa*, also known as blackseed oil, has been shown to exert anti-viral effects (Barakat et al., 2013; Onifade et al., 2013; Onifade et al., 2015). *Nigella Sativa* oil was associated with faster recovery in an open label randomized clinical trial (Koshak et al., 2021b). Moreover, a multicenter randomized clinical trial showed that a honey and *Nigella Sativa* combination resulted in a significant reduction in the severity of clinical symptoms, earlier viral clearance, and reduced mortality in COVID-19 patients (Ashraf et al., 2020).

TQF (NP-101) is a novel patent-pending, enteric-coated oral formulation, derived from *Nigella Sativa*, with TQ being its main ingredient. Our group has recently conducted a randomized double-blind, placebo-controlled clinical trial with TQF. The primary endpoints of this study were safety and the median time-to-sustained-clinical-response (SCR).

SCR was 6 days in the treatment arm vs. 8 days in the control arm (*p* = 0.77). Importantly, SCR was 5 days in the treatment vs. 7.5 days in the placebo arm in the high-risk patients; HR 1.55 (95% CI: 0.70, 3.43, *p* = 0.25). High-risk features were defined based on the presence of at least one of the following risk factors: age≥60, obesity, diabetes mellitus, hypertension, chronic cardiopulmonary disease, or auto-immune disease, which were selected based on similar high-risk features reported in recent COVID-19 studies. Notably, we did not observe a significant rate of adverse event differences between the two arms (*p* = 0.16). Furthermore, in this study, TQF-treated patients were found to have a significantly faster decline in their TSB (*p* < 0.001), and our biomarkers data showed a significant increase in the treatment *versus* placebo arm in cytotoxic CD8^+^ (*p* = 0.042) and helper CD4^+^ (*p* = 0.042) central memory T lymphocytes. Although there was no statistically significant difference in SCR between the TQF and placebo arms, SCR was shorter in the treatment arm, especially in high-risk patients and TQF activities across multiple endpoints were significant. Therefore, a large, randomized phase 2 double-blind, placebo-controlled study in high-risk outpatient COVID-19 patients is planned accordingly. Importantly, pre-clinical studies showed that TQ and TQF inhibit the entry and infection of five SARS-CoV-2 variants by blocking the ACE2 receptor (Bencheqroun et al., 2022). This is very relevant to COVID-19 patients since ACE2 expression has been found to be closely related to morbidity and mortality of COVID-19 infection (Chaudhry et al., 2020; Ni et al., 2020; Obukhov et al., 2020; Chen et al., 2021; Kaseb et al., 2021).

Free fatty acids, such as arachidonic acid, oleic acid, and linoleic acid, have inactivated enveloped viruses, such as influenza and herpes (Kohn et al., 1980a; Kohn et al., 1980b). Polyunsaturated fatty acids, such as linoleic acid, have exerted antimicrobial effects by enhancing the generation of free radicals, augmenting the formation of cytotoxic lipid peroxides, and by increasing the formation of their bioactive metabolites (Das, 2018). Here we show that linoleic acid, oleic acid, and palmitic acid have an inhibitory effect on SARS-CoV-2 pseudovirus infection at non-toxic concentrations. The exact mechanism of the inhibition is currently unknown. However, it has been shown that polyunsaturated fatty acids including linolenic acid effectively interfered with binding to hACE2 in a dose-dependent manner (Goc et al., 2021). The same study further demonstrated that linolenic acid and eicosapentaenoic acid showed a significant direct inhibitory effects on the activity of the host proteases TMPRSS2 and cathepsin L in addition to inhibiting viral binding (Goc et al., 2021). Additionally, in observing the relative infectivity response of pseudo-viruses 614G and Omicron to incremental TQ formula doses showed a similar pattern of decline to both subvariant viruses, despite any differences in their mutation profiles. Both had a particularly sudden dose response point, TQ, at .01%, and sharply declined the infectivity for both the subvariants. Evident to the data, the fatty acid, TQ formulation, has an effective inhibitory effect on SARS-CoV-2 viruses.

We also tested combinations of fatty acids and TQ to mimic the effect of novel NP-101. Especially palmitic acid showed a strong inhibition when combined with TQ or other fatty acids. However, this inhibitory effect differed for the Delta, 614G, United Kingdom, and Brazil COVID-19 variants, with the strongest inhibition observed for the Delta variant and the weakest inhibition observed for the Brazil variant. These findings were unexpected, and at this time we do not have a clear explanation for them. It is possible that the varied levels of inhibition could be partly due to different mutational profiles in the receptor-binding domain (RBD) that each variant carries. The Brazil variant bears three mutations (K417T, E484K, and N501Y) in the RBD that is thought to enhance the binding affinity of the spike protein for ACE2 (Gu et al., 2020; Starr et al., 2020; Villoutreix et al., 2021). In contrast, the Delta variant also carries a L452R mutation in the receptor binding site which appears to increase the interaction between RBD and the ACE-2 and infectivity (Kirola, 2021). Although different studies have shown different binding affinities between RBD and ACE, one group reported that the United Kingdom variant (alpha) had a 10-fold increase, the Brazil variant (gamma) had a 5-fold increase, and the Delta variant had a 2-fold increase in ACE binding affinity compared to the ancestral RBD (Liu et al., 2022).

Our study has some limitations, including the lack of *in-vivo* studies and the exact mechanism of inhibiting ACE2 enzymatic activity. However, in line with this literature, we also showed that oleic, palmitic, and linoleic acid had an inhibitory effect on SARS-CoV-2 pseudovirus. Importantly, polyunsaturated fatty acids, particularly linolenic acid, eicosapentaenoic acid, and linoleic acid, have also been shown to inhibit SARS-CoV-2 binding and entry by interfering with binding to the human ACE receptor (Goc et al., 2021). Finally, our promising signals from the recently completed small randomized phase 2 study acted as a significant proof of concept of the effects of NP-101 on COVID-19 in the outpatient setting (Bencheqroun et al., 2022).

## 5 Conclusion

This study, we showed that not only TQ but also other individual ingredients of NP-101, such as palmitic, oleic, and linoleic acids had inhibitory effects on SARS-CoV-2 variants with various efficacy, possibly by inhibiting viral entry via the ACE-2 receptor. Further *in vivo* experiments are warranted to validate the findings of this study. Accordingly, a large, randomized phase 2 study is planned in high-risk COVID-19 patients.

## Data Availability

The raw data supporting the conclusions of this article will be made available by the authors, without undue reservation.
