# Supplementary material for: Individual ingredients of NP-101 (Thymoquinone formula) inhibit SARS-CoV-2 pseudovirus infection
Source: Front Pharmacol. 2024 Feb 6;15:1291212. doi: 10.3389/fphar.2024.1291212 (PMC10876831; doi:10.3389/fphar.2024.1291212)
Supplement: Supplementary file 1 [file DataSheet1.docx]

**Supplementary Materials:** Supplementary figure 1-5

**Supplementary figure 1: Plasmid map of SARS-CoV-2-Spike expression vector**

**
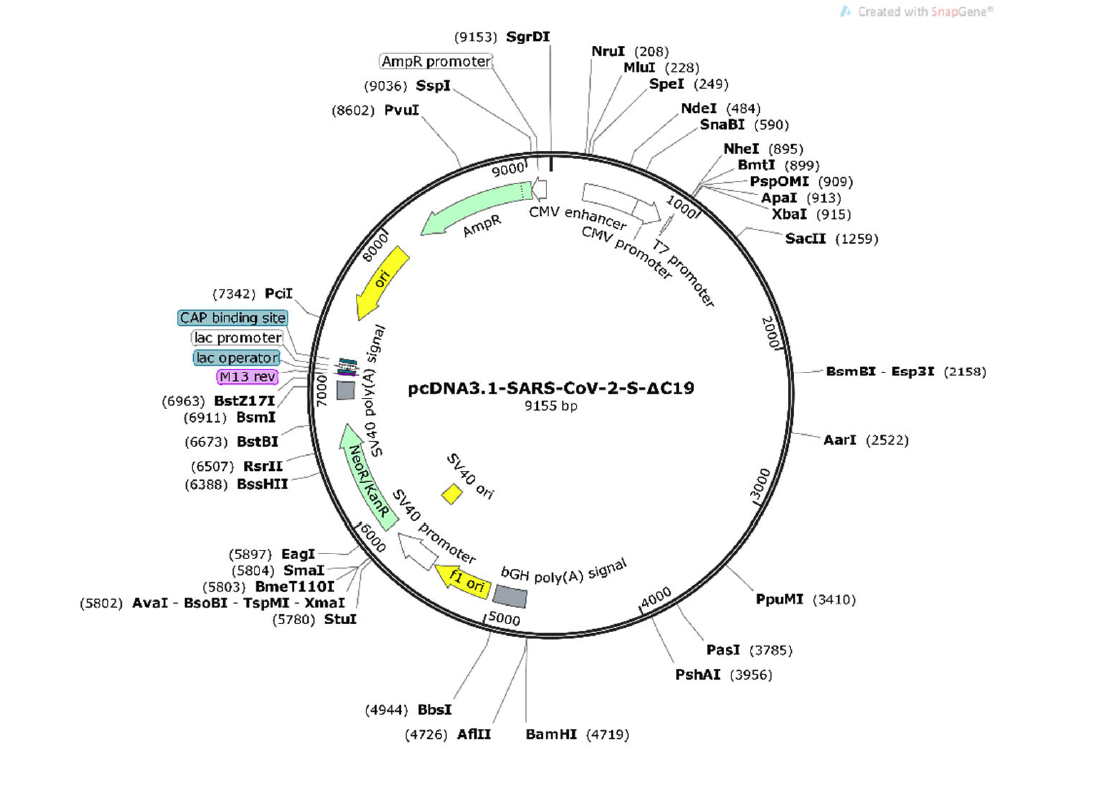
**

**Supplementary Fig. 2 Protein sequence of SARS-CoV-2-614G Spike-ΔC19**

**
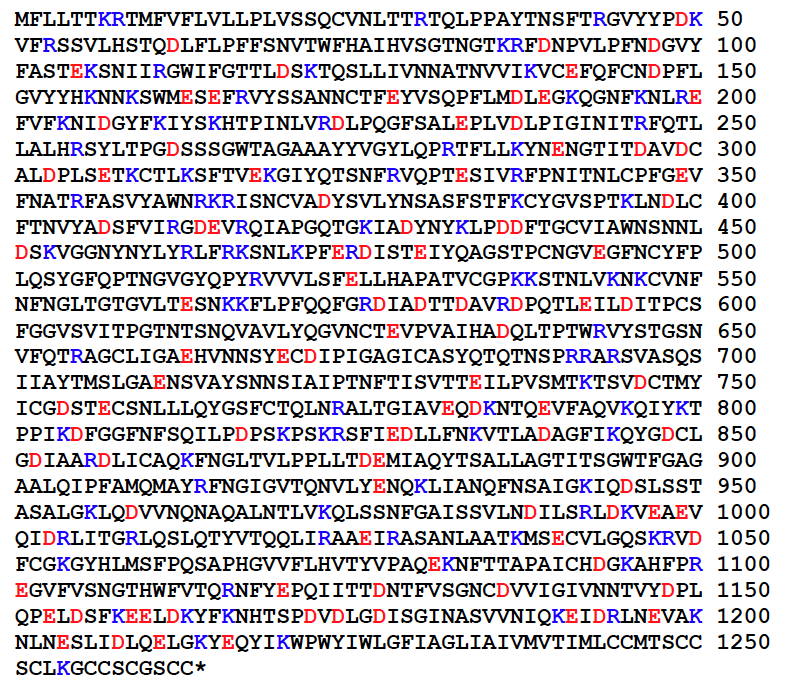
**

**Supplementary Fig. 3 Protein sequence of SARS-CoV-2-UK variant Spike-ΔC19**

**
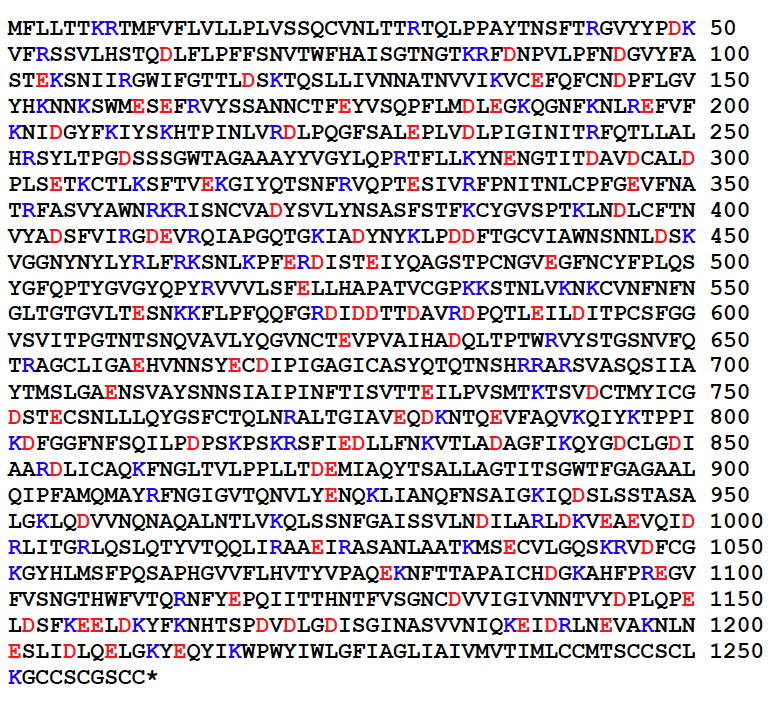
**

**Supplementary Fig. 4 Protein sequence of SARS-CoV-2-Delta variant Spike-ΔC19**

**
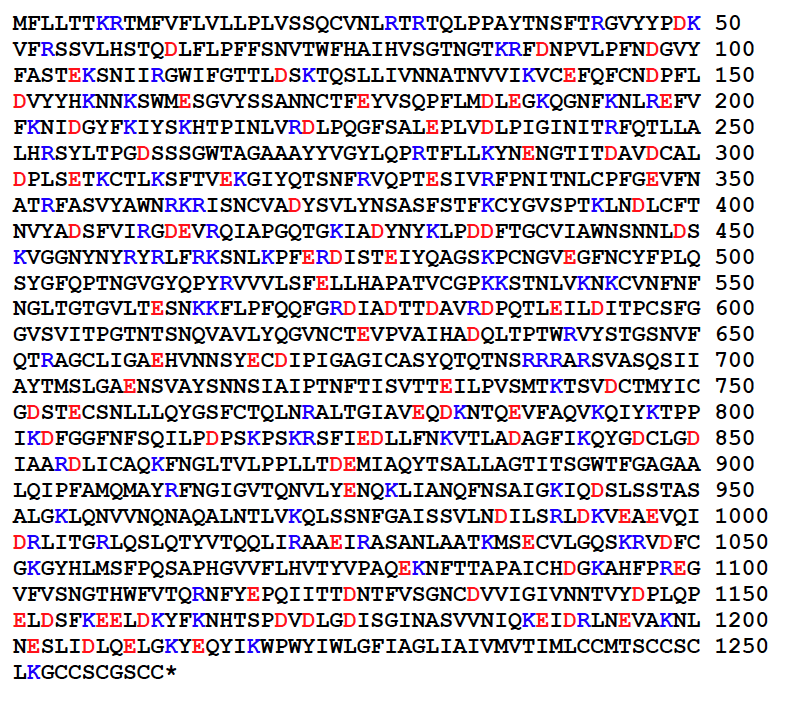
**

**Supplementary Fig. 5 Protein sequence of SARS-CoV-2-Brazil variant Spike-ΔC19**

**
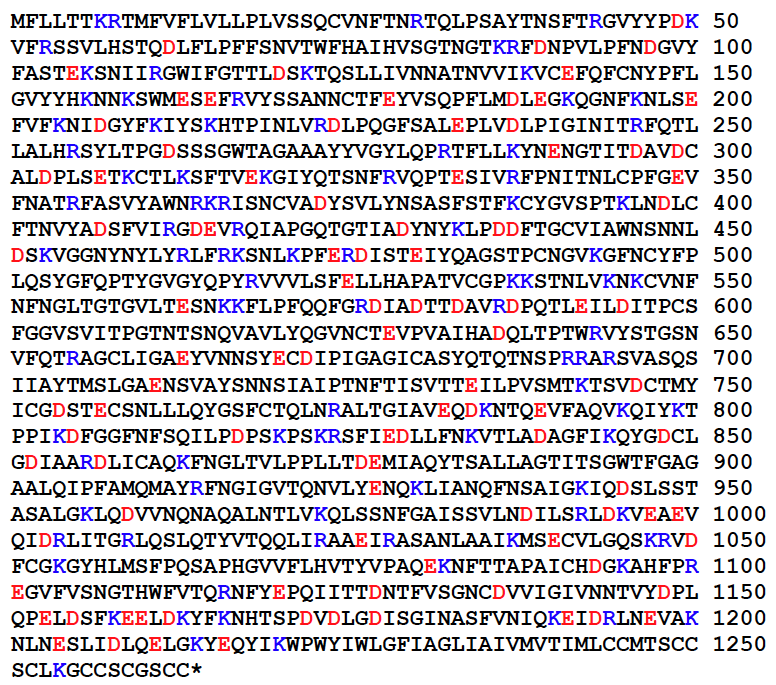
**
